# Case report: Intrapleural plus systemic Tislelizumab injection combined chemotherapy in RET gene fusion-positive lung adenocarcinoma presenting refractory malignant pleural effusion

**DOI:** 10.3389/fonc.2024.1404173

**Published:** 2024-09-20

**Authors:** Dong Qiu, Xiao-Hui Zhang, Yang Wang, Cheng Chen

**Affiliations:** Department of Respiratory and Critical Medicine, The First Affiliated Hospital of Soochow University, Suzhou, China

**Keywords:** intrapleural immunotherapy, immunotherapy, RET gene fusion, lung adenocarcinoma, malignant pleural effusion

## Abstract

RET fusions were discovered in non-small cell lung cancer (NSCLC), and the efficacy of RET-targeted treatment in these patients has been previously established. However, patients with required resistance to RET-TKIs have limited treatment options. Herein, we describe a case of critical and advanced lung adenocarcinoma harboring RET fusion. Despite a significant response to Prasetinib previously, the patient developed refractory malignant pleural effusion after 24 months of treatment. He was treated simultaneously with intrapleural plus systemic Tislelizumab injection combined chemotherapy, thereby achieving an excellent clinical benefit maintaining control of pleural effusion for over 6 months. Hence, we would like to discuss intrapleural immunotherapy as an additional treatment method in refractory malignant pleural effusion while demonstrating good treatment tolerance.

## 1 Introduction

Rearranged during transfection (RET) is a proto-oncogene that belongs to the receptor tyrosine kinase (RTK) family. Previous studies have demonstrated that RET rearrangements contribute to cellular survival, migratory capacity, and proliferative potential. In 2012, RET fusions were initially detected in non-small cell lung cancer (NSCLC), which are present in 1%–2% of lung cancer patients (1).

The treatment of lung cancers harboring a RET fusion has evolved from traditional chemotherapy towards targeted therapy with selective RTK inhibitors and immunotherapy. The therapeutic effects of different RET fusion partner genes exhibit significant diversity. Therefore, the precise selection of RTK inhibitors is particularly significant in clinical practice. Pralsetinib demonstrates significant efficacy against RET fusion genes. However, tumor recurrence remains a frequent clinical challenge due to acquired drug resistance (2, 3).

Currently, the mechanisms of resistance to these selective RET-TKIs are being studied. Recurrent resistance mechanisms include RET solvent front mutations and MET amplification, while KRAS amplification has also been identified in resistant cases (4, 5). Moving forward, it will be important to develop next-generation RET inhibitors and other treatment option such as combination strategies, to overcome resistance and improve their prognosis with *RET* fusion-positive lung cancer.

Patients suffering from malignant pleural effusion (MPE) are associated with high mortality and exhibit a grim prognosis (6). The appearance of MPE in patients on targeted drugs usually indicated recurrence of lung cancer, which was signaled that the disease had persisted in progressing after receiving prior treatment. These patients needed a second-line treatment to control the progression (7). Conventional clinical treatment for MPE mainly includes chest drainage, pleural fixation, and intrathoracic drug infusion (8). Cisplatin is widely used in intrathoracic drug infusion, but resistance may occur after multiple doses. With the advances in antitumor drugs, a growing number of new drugs are being applied in clinical practice; for example, antiangiogenic drugs are widely used in recent years (8). The main antiangiogenic drugs are bevacizumab and recombinant human vascular endothelial growth factor (VEGF) inhibitors, both of which can be administered via thoracic perfusion. The combination of these antiangiogenic drugs with platinum agents has proven to be more effective than platinum agents or antiangiogenic drugs alone. This synergistic effect leads to enhanced inhibition of tumor cell growth and a more effective reduction in effusion formation (9).

In the past few years, there was an attempt to use immune checkpoint inhibitors to improve MPE by intrathoracic injection. Previous research has demonstrated that intrathoracic injection anti-programmed cell death protein 1 (PD1) has significantly reduced pleural effusion and extended the survival time of mice. Correspondingly, intrathoracic injection of anti-PD1 mAb improved the activity and function of cytotoxic T cells (CTLs) in the local tumor microenvironment of MPE (10). Intrapleural injection of PD1 mAb has proven to be effective in managing MPE in mouse models and has also shown promising results in a small-scale human study, but no study was reported in non-small cell lung cancer patients with RET acquired resistance.

Herein, we present an interesting clinical case of a critical lung adenocarcinoma harboring RET fusions. This patient initially received salvage Pralsetini therapy and exhibited a prolonged remission for 24 months. Inevitably, tumor progression was eventually noted on a further follow-up CT scan but showed a rapid response to integrated intrapleural plus systemic Tislelizumab injection combined chemotherapy.

## 2 Case presentation

**Figure 1:**
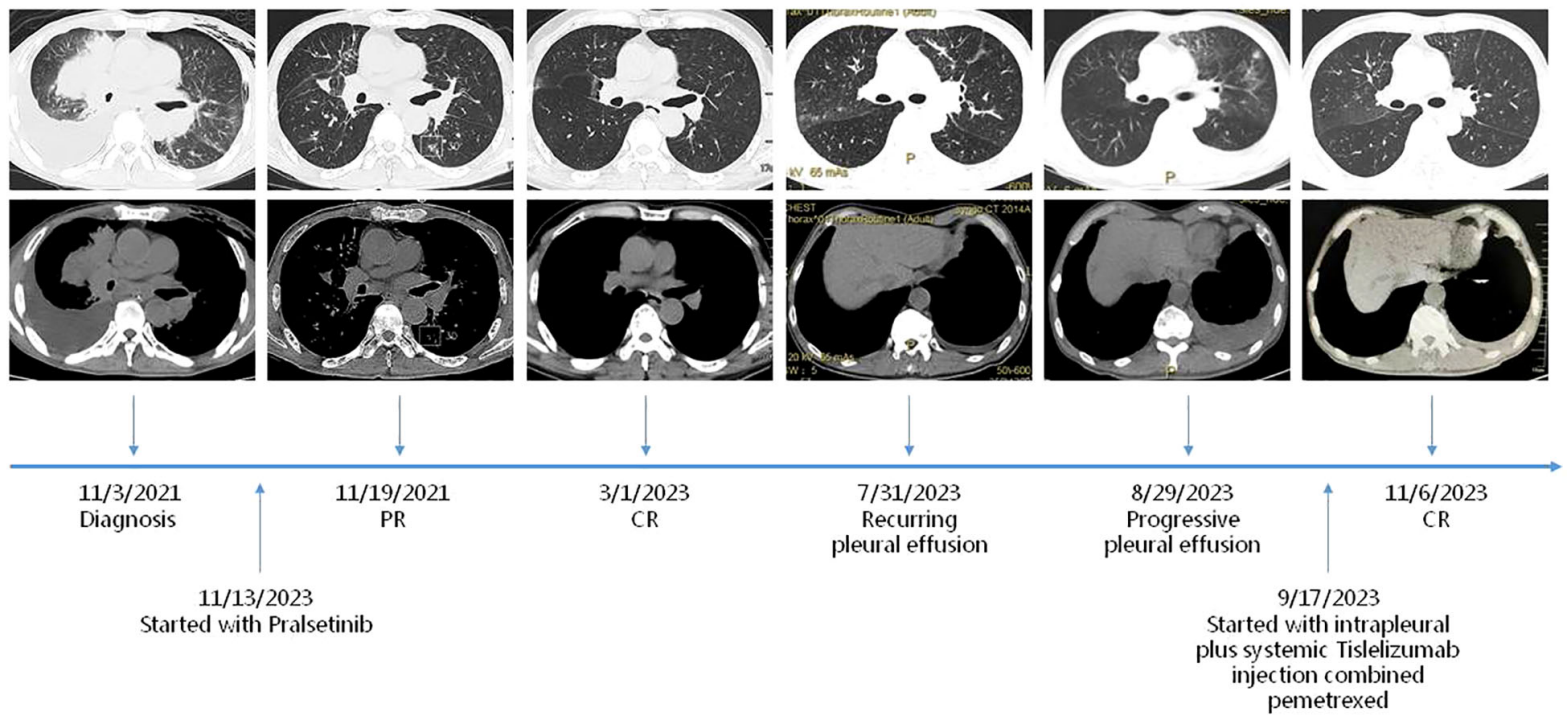
Timeline. Maximum diameters of lesion at diagnosis on 3/11/2021, at PR (partial remission) on 19/11/2021, at durable CR (complete remission) on 1/3/2023, at PD (progressive disease) on 31/7/2023 and 29/8/2023, and at PR (partial remission) on 6/11/2023.

We present a case of a 55-year-old man who was diagnosed with stage IV NSCLC in November 2021 (**Figure 1**). This patient had no history of chronic illness, no familial lung cancer history, and no familial genetic disease. He had a history of smoking for 30 years. Initially, the patient presented with progressive dyspnea, exercise intolerability, and recurrent chest pain. For the exacerbation, he was admitted to the emergency department in late October 2021. A chest CT scan revealed a neoplastic lesion in the right hilum, with a maximum diameter of approximately 8 cm × 7 cm, accompanied by mediastinal lymphadenopathy and pleural effusion. Due to cardiac arrest, he received cardiopulmonary resuscitation and mechanical ventilation. Extensive VTE was noticed at the same time. The histology of the tumor was obtained by draining pleural effusion from the right lung. Pathological examination revealed lung adenocarcinoma, and the initial staging of the patient resulted in UICC IV. Genetic mutations in tumor tissues were tested. This analysis revealed a RET fusion. No ALK, ROS1, EGFR, or NTRK1/2/3 fusion transcripts and no MET amplifications were found. The newly acquired sample revealed that the PD-L1 status could be evaluated to reach up to 30% on tumor cells. After multimodality therapy, the patient recovered and had been spontaneously breathing without respirator. He was globally configured as PS 4. As the patient furthermore suffered from severe VTE and respiratory failure, coupled with the fact that NSCLC patients harboring oncogenic driver mutations typically respond rapidly to targeted therapies, we decided to initiate TKI therapy with Pralsetini at the mid-month of November 2021, starting with 400 mg taken orally once daily. After 1 week of treatment, the maximum diameter of the neoplastic lesion in the right hilum had decreased from 8 cm ×7 cm to 3 cm ×2.5 cm. We conducted a CT follow-up examination after 40 days of treatment; the neoplastic lesion disappeared, leaving a cancerous cavity, and the size of metastatic cancerous nodules shrank, which revealed overall therapeutic effect to Pralsetini therapy. The patient’s symptoms had completely relieved, and he was globally configured as PS 0. Follow-up CT scans were conducted in 2022 and 2023, which showed stable disease based on Response Evaluation Criteria in Solid Tumors (RECIST). Additionally, the patient continued to tolerate Pralsetini without any drug toxicity.

However, the patient again developed a progressive pleural effusion along with a progression of pulmonary nodule in July and August 2023. NSCLC cells were found in the pleural effusion. Globally, it was configured a PS 1. The patient was not competent for his job because of declined habitual physical activity. Since the patient has not undergone immunotherapy or chemotherapy before, the decision was made for a chemoimmunotherapy in this setting. The therapy was initiated in late September 2023 and consisted of Pemetrexed 500 mg/m^2^ and Tislelizumab 200 mg. As malignant pleural effusion is a major therapeutic challenge and associated with deteriorated quality of life and elevated mortality rates, we then exploited intrapleural Tislelizumab 100 mg injection as an adjunctive therapy alongside all systemic treatments. The patient tolerated the administration well and did not show any clinical symptoms of side effects or toxicity during the first follow-up. In addition, we conducted a CT follow-up examination, which revealed global therapeutic response of the cancer to this therapy, together with regressed pleural effusion. As the patient consistently showed good treatment tolerance, further rounds of chemotherapy and immunotherapy were administered, maintaining the same dosage throughout. The patient has resumed normal life and work, who was configured a PS 0. After every two rounds of treatment, we conducted follow-up CT scans as a monitoring indicator. The CT scan revealed a stable reduction in pleural effusion over 6 months, with the response maintained and continued to persist.

## 3 Discussion

Here, we describe an interesting case report on a patient with extensive VTE involvement due to NSCLC and PS >4 in which treatment with Pralsetini achieved an excellent clinical benefit. Upon acquired resistance, simultaneous treatment with intrapleural plus systemic Tislelizumab injection combined chemotherapy generated quick and obvious response to NSCLC. As a supplement, the use of Pralsetini as initial treatment for this patient is similarly debatable. Our decision was to refrain from his critical status involved from NSCLC and potential quick response to targeted therapies among NSCLC patients harboring oncogenic driver mutations.

Until recently, the treatment of RET fusion-positive NSCLC lacks specific guidelines when resistance to targeted therapy developed. Even for first-line treatment, standard platinum-based chemotherapy is associated with moderate response rates (15%–41%) and short PFS (median, 4.5–6.5 months). Outcomes with immune checkpoint inhibitors also remain poor for these patients (ORR of 0%–7% and median PFS of 2.2–3.4 months), including those positive for PD-L1 expression (≥1% PD-L1). Otherwise, the majority of NSCLC patients harboring RET fusions exhibit low levels of PD-L1 expression and tumor mutation burden, resulting in suboptimal responses to immune checkpoint inhibitors (11).

According to the IMPOWER150, regimen comprising of Carboplatin, Paclitaxel, Bevacizumab, and Atezolizumab would have been a viable therapeutic option to third EGFR-TKI-acquired resistance (12). Hence, immunotherapy combined with chemotherapy would have been justifiable in this case. However, the landscape of management options to improve outcomes for the patient remains limited.

Given that refractory MPE acted as the feature in this case, the treatment of pleural malignancies remains a significant challenge, as they often exhibit limited responsiveness to conventional treatment, including systemic chemotherapy and immunotherapy (13, 14). Previously, local intrapleural chemotherapy could be administered to patients with MPE to eliminate tumor cells directly while also promoting pleural adhesions to minimize pleural fluid recurrence. However, there is a scarcity of studies that evaluate the significance of intrapleural chemotherapy in the treatment of advanced primary lung cancer. Advancements in intrapleural immunotherapy have demonstrated overall good tolerance with variable clinical responses. Vital categories of intrapleural immunotherapies encompass intrapleural cytokines, innate immunomodulatory agents, cellular immunotherapies, and viral-vector-mediated delivery of immunostimulatory and/or oncolytic genes (14, 15). Considering the potential for stimulating tumor-specific immune responses in the pleural space of patients with MPE, intrapleural injection of PD1 mAb has been an area of significant interest. This approach has demonstrated effective control of MPE in mouse models and a limited human study. Cellular immunity, with CD8+T cells as pivotal mediators, is crucial in anti-tumor response. CD8+T cells are primarily categorized into four main subsets: naive cells (Tnaive), central memory cells (Tcm), effector memory cells (Tem), and terminally differentiated effector memory cells (TemRA). Previous research demonstrated that MPE exhibits significantly higher percentages of CD8+T cells compared to peripheral blood (PB), with fewer TemRA/Tnaive but more Tem/Tcm. MPE-Tem and MPE-Tcm secreted more IFNγ, TNFα, and IL-2 compared to paired MPE-TemRA and MPE-Tnaive, which were used to kill tumor cells. However, inhibitory molecules (PD-1, Tim-3, CD39, and CTLA-4) in MPE caused damage to CD8+T cells. A novel immunotherapeutic strategy could involve administering antibodies directed against PD1 or CD39 via intrapleural injection (16). However, large-scale studies evaluating the safety and effectiveness of intrapleural immunotherapy are still awaited.

In the present case, we exploited intrapleural Tislelizumab 100 mg injection as a supplementary therapy in conjunction with all systemic treatments. Based on the short-term CT follow-up examination, a comprehensive therapeutic response of the NSCLC to this therapy, together with regressed pleural effusion, was revealed. As supported, the pleural space in NSCLC represents an extension of the tumor microenvironment, and PD-1 mAb can serve as a potent stimulatory agent to counteract the tumor-mediated immune tolerance and T-cell exhaustion. The proposed benefits of intrapleural PD-1 mAb injection include site-specific concentration of therapeutic agents together with direct contact with tumor cells. In addition, the administration of systemic and intrapleural immunotherapy was expected to alter the immune composition of effusions in patients with MPE, thus producing synergistic therapeutic effect. Next, we will set up a double-blind controlled cohort to compare PD-1 mAb vs. talcum powder intrapleural injection efficacy.

In conclusion, the optimal treatment approach for this particular setting of RET-TKIs-resistant lung adenocarcinoma remains undecided and might also depend on individual patient characteristics. We analyzed our case comparing with currently published data about treatment of RET-targeted drug resistance in NSCLC, even though there is no published experience about the integration of three treatments. This case contributes to propose intrapleural immunotherapy as a feasible treatment option, and intrapleural PD-1 mAb bridging to systemic immunotherapy may represent a promising approach in NSCLC treatment. As with other potential applications of immunotherapy, immune biomarkers need to be better understood to aid in selection of this therapy.

## Data Availability

The original contributions presented in the study are included in the article/supplementary material. Further inquiries can be directed to the corresponding author.
